# Safety and Feasibility of Interventional Hybrid Fluoroscopy and Nuclear Imaging in the Work-up Procedure of Hepatic Radioembolization

**DOI:** 10.1148/rycan.240044

**Published:** 2024-11-01

**Authors:** Martijn M. A. Dietze, Marjolein B. M. Meddens, Rob van Rooij, Arthur J. A. T. Braat, Bart de Keizer, Rutger C. G. Bruijnen, Marnix G. E. H. Lam, Maarten L. J. Smits, Hugo W. A. M. de Jong

**Affiliations:** From the Department of Radiology and Nuclear Medicine, University Medical Center Utrecht, Heidelberglaan 100, 3584 CX, PO Box 85500, 3508 GA Utrecht, the Netherlands.

**Keywords:** Angiography, Fluoroscopy, Interventional-Vascular, Radionuclide Studies, Radiosurgery, Gamma Knife, Cyberknife, SPECT, Instrumentation, Physics, Technical Aspects, Technology Assessment

## Abstract

**Purpose:**

To evaluate the safety and feasibility of a novel hybrid nuclear and fluoroscopy C-arm scanner to be used during the work-up procedure of hepatic radioembolization.

**Materials and Methods:**

In this prospective first-in-human clinical study, 12 participants (median age, 67 years [range: 37–78 years]; nine [75%] male, three [25%] female) with liver tumors undergoing work-up for yttrium 90 radioembolization were included (ClinicalTrials.gov NCT06013774). Work-up angiography and technetium 99m–macroaggregated albumin injection were performed in an angiography suite equipped with a hybrid C-arm that could simultaneously perform fluoroscopy and planar nuclear imaging. Technetium 99m–macroaggregated albumin was injected under real-time hybrid imaging, followed by in-room SPECT imaging. Safety and feasibility were studied by assessing adverse events, technical performance, additional x-ray radiation dose, and questionnaires completed by radiologists and technologists.

**Results:**

No adverse events were attributed to the hybrid C-arm scanner. The additional x-ray radiation dose was low (median, 19 Gy · cm^2^; minimum: 12 Gy · cm^2^; maximum: 21 Gy · cm^2^ for participants who completed all imaging steps). The interventional personnel considered use of the hybrid C-arm scanner safe and feasible, although the additional time spent in the intervention room was considered long (median, 64 minutes; minimum: 55 minutes; maximum: 77 minutes for participants who completed all imaging steps).

**Conclusion:**

Use of the hybrid C-arm scanner during the work-up procedure of hepatic radioembolization was found to be safe and feasible in this first-in-human clinical study.

**Keywords:** Angiography, Fluoroscopy, Interventional-Vascular, Radionuclide Studies, Radiosurgery, Gamma Knife, Cyberknife, SPECT, Instrumentation, Physics, Technical Aspects, Technology Assessment

*Supplemental material is available for this article.*

Published under a CC BY 4.0 license.

Clinical trial registration no. NCT06013774

**Summary**

Use of a hybrid C-arm scanner, which performs simultaneous fluoroscopy and nuclear imaging, was found to be safe and feasible during the work-up procedure of hepatic radioembolization.

**Key Points**

■ This first-in-human study demonstrated the safety and feasibility of using the hybrid C-arm scanner for simultaneous fluoroscopy and nuclear imaging in 12 participants undergoing a work-up procedure for hepatic radioembolization, with no adverse events attributed to use of the hybrid scanner and low additional radiation dose (median, 19 Gy · cm^2^) per participant.
■ Images acquired during the procedures demonstrated the potential applications of the device for image guidance of interventional procedures involving radionuclides.

## Introduction

C-arm fluoroscopy uses x-rays to acquire real-time planar and three-dimensional images during interventional procedures. Hybrid imaging, such as fluoroscopy together with scintigraphy, may offer added value for guiding procedures in which radiopharmaceuticals are or may be used (eg, radioguided surgery, biopsies, intra-arterial radionuclide therapy, cardiac interventions) (1). Similar applications of hybrid imaging have been successfully developed for PET/CT interventional image guidance (2,3).

Previously, a C-arm scanner was developed that can perform hybrid fluoroscopic and scintigraphic imaging in the intervention room, and its performance has been tested using phantom experiments (4–6). The transition to using this hybrid C-arm scanner in a clinical setting poses several challenges given the hectic environment of the intervention room, the complexity of changing the existing workflow for interventional procedures, and the large number of personnel who must adapt to the new technology.

Therefore, the aim of this first-in-human study was to evaluate the safety and feasibility of the hybrid C-arm scanner in a clinical setting. The scanner was tested in the context of hepatic radioembolization (7,8) because this is a promising application for hybrid interventional imaging. The conventional radioembolization workflow consists of three steps. First, an angiographic procedure is performed to study the patient’s liver vasculature and to inject technetium 99m–macroaggregated albumin (^99m^Tc-MAA) particles at specific injection sites. Second, the patient is transferred to the nuclear medicine department and receives a SPECT scan to visualize the ^99m^Tc-MAA distribution in the liver and surrounding organs. The images show whether the ^99m^Tc-MAA particles primarily end up in tumorous tissue and not inadvertently in healthy tissue. If a promising distribution is achieved (ie, good tumor targeting), microspheres are ordered for the treatment. If a bad distribution is obtained (eg, bad tumor targeting or major particle accumulation in organs other than the liver), the patient is not eligible for treatment. Third, the patient receives treatment by undergoing a second angiographic procedure in which the microspheres are injected according to the treatment plan. With a hybrid imaging device as described in this work, the above three steps may be merged into a single-session treatment, saving time, costs, and personnel as well as potentially improving patient comfort and treatment outcome.

## Materials and Methods

### Study Design

This first-in-human study (ClinicalTrials.gov NCT06013774) performed at the University Medical Center Utrecht (Utrecht, the Netherlands) followed a three-plus-three clinical escalation study design. The study was approved by the University Medical Center Utrecht institutional review board. The prospective study consisted of three cohorts, with participants enrolled between January 2021 and May 2023. All participants provided written informed consent before study participation. In the first cohort, the safety of planar imaging was assessed. In the second cohort, the safety of planar imaging and three-dimensional imaging was assessed. In the third cohort, the safety of planar imaging and three-dimensional imaging was assessed and more information on the feasibility was retrieved.

If one procedure within one cohort was deemed unsafe, three additional participants were enrolled. If two procedures within one cohort were deemed unsafe, the study was terminated.

In total, at least 12 participants were scheduled to be included in the study. If the first two cohorts of three participants successfully completed the study, the third cohort consisted of six participants. If one of the first two cohorts was extended, the third cohort consisted of three participants.

With 12 to 15 participants, the study sample was expected to have a reasonable spread in male-female distribution, body mass index, length, and overall physical condition to evaluate the device’s performance under realistic clinical conditions.

### Participants

Patients with liver tumors who were scheduled for yttrium 90 (^90^Y) radioembolization work-up as decided by the multidisciplinary tumor board were eligible for study inclusion. Exclusion criteria included individuals who were insufficiently fit to undergo additional examination time of 30–90 minutes (to not overly burden individuals in poor health who would gain no immediate benefit from the study), patient whose height was greater than 190 cm or bust line was greater than 135 cm (to fit the scanner geometry), and more than two expected ^99m^Tc-MAA injection positions (to limit the total time of the procedure). An individual’s participation in the study ended on acquisition of the last scan using the hybrid C-arm scanner.

### Hybrid C-Arm Scanner

**Figure 1:**
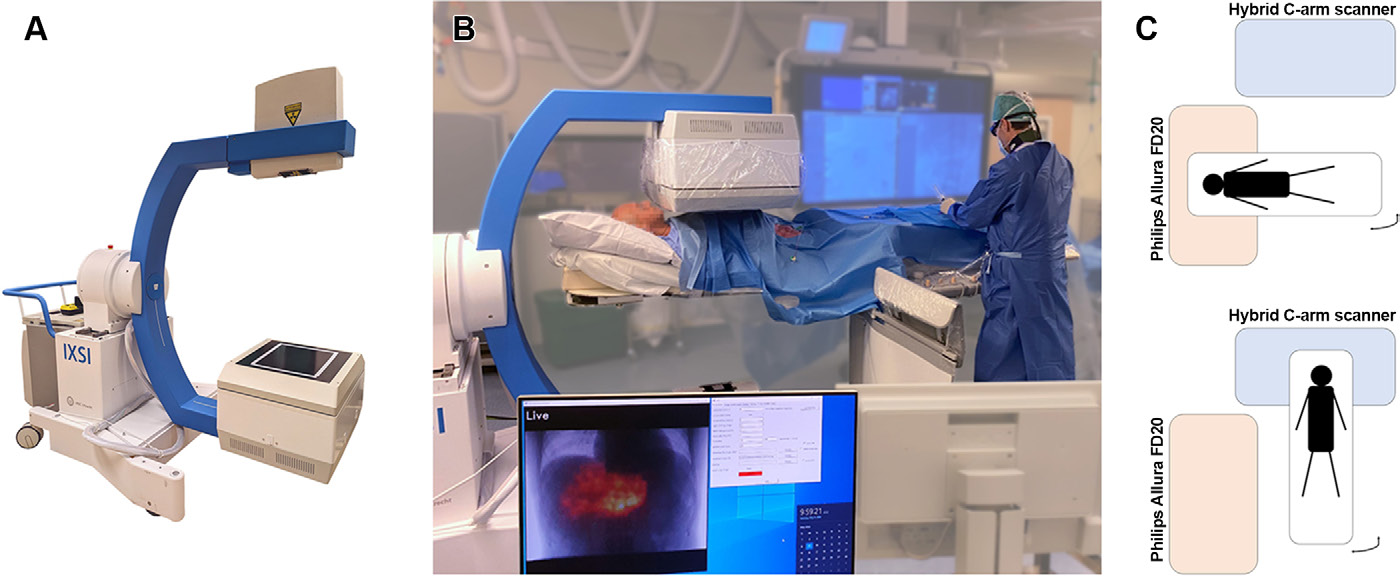
**(A)** Hybrid C-arm scanner used in this study. **(B)** Typical example of the hybrid C-arm scanner used during radioembolization work-up in the intervention room. **(C)** Schematic illustration of the placement of the hybrid C-arm scanner in relation to the clinical cone-beam CT system. The patient table is rotated 90 degrees to switch imaging with both scanners.

The hybrid C-arm scanner (named IXSI: Interventional X-Ray and Scintigraphy Imaging, see Fig 1A) comprised an x-ray tube (from a Philips Veradius System) placed on a C-arm gantry on the opposite side of a dual-layer detector. The C-arm gantry was manufactured to be compact and mobile so that the scanner could be used in multiple operation rooms. The dual-layer detector consisted of a flat panel x-ray detector placed in front of a gamma camera with a cone-beam collimator. The cone-beam collimator had holes with 40.0-mm length, 1.90-mm inner diameter, and 0.25-mm septal thickness. The 39.9 × 29.5-cm^2^ flat panel detector (no antiscatter grid) was adapted from a clinical Philips Allura C-arm system to be relatively transparent to 140-keV photons (average gamma transmission of 52% at 140 keV) and had a thickness of 6.8 cm. The gamma camera was custom made (Intermedical) and equipped with short photomultiplier tubes and optimized shielding. The 51.0 × 38.1-cm^2^ gamma camera had a 9.5-mm-thick thallium-doped sodium iodide scintillation crystal with 3.9-mm full width at half maximum intrinsic spatial resolution and 9.4% energy resolution at 140 keV. For imaging of ^99m^Tc at 10-cm distance, the gamma camera had a sensitivity of 66.2 cps/MBq and a full width at half maximum resolution of 12.1 mm. The x-ray tube was placed at approximately the focal spot of the cone-beam collimator (105-cm distance) so that x-ray and nuclear images were intrinsically registered. The x-ray tube allowed beam strengths between 40 and 80 kVp and between 0.02 and 14.00 mA and pulses between 3 and 15 Hz. A dose area product meter in the housing of the x-ray tube monitored the delivered radiation dose. The custom mobile C-arm gantry allowed for flexible two-dimensional imaging as well as three-dimensional cone-beam CT (CBCT) and SPECT with programmable noncircular orbit following the outline of the participant. The dual-layer detector was equipped with a detection system that terminated motion upon mechanical compression (eg, upon collision with the participant). The scanner is an experimental device (not available on the market) and was certified by the local medical ethics committee to be used in clinical trials within the institute. Dietze et al (5) provide further details on the technical fluoroscopic and nuclear imaging performance.

### Study Workflow

Participants underwent a routine work-up procedure, meaning that the positioning of the catheter was performed on a clinical fluoroscopy and CBCT system (Allura Xper FD20; Philips). When the interventional radiologist considered the catheter to be in the desired position for the first ^99m^Tc-MAA injection, the patient table was rotated by 90 degrees so that the participant was in the field of view of the hybrid C-arm scanner (see Fig 1B). A digital subtraction angiography image was acquired and compared with the digital subtraction angiogram acquired on the Philips system to ensure that the catheter had not moved during the table rotation. The procedure continued on confirmation of the correct catheter location (otherwise, the patient table was rotated back and the catheter repositioned).

Next, ^99m^Tc-MAA was injected during hybrid imaging. For this task, an adjustment was made to the regular protocol: The syringe that normally contained 100 MBq ^99m^Tc-MAA was split over four syringes of approximately 25 MBq each, which were injected relatively slowly at intervals of approximately 1 minute. This protocol adjustment was performed to determine whether the distribution of ^99m^Tc-MAA changed as more particles were injected (eg, due to embolization). In the case of multiple injection positions, only the injection at the first position was dynamically visualized with the hybrid C-arm scanner to limit the discomfort of the participant (time per injection position was approximately 10 minutes). Imaging was performed at the first injection position to ensure there was no background gamma radiation component in the images, allowing for good visualization of the ^99m^Tc-MAA build-up. Once all ^99m^Tc-MAA was injected, the hybrid C-arm scanner acquired a 10-minute SPECT/CBCT acquisition of the liver region and 1-minute anterior and posterior static planar acquisitions of the lung and liver regions. The interventional radiologists (R.C.G.B., with 18 years of experience, and M.L.J.S., with 13 years of experience) were blinded to the acquired images for the duration of the study.

Typical x-ray settings with IXSI were 65 kVp with 10.8 mA at 15 Hz for digital subtraction angiography imaging, 65 kVp with 0.4 mA at 4 Hz for fluoroscopy, and 70 kVp with 1.2 mA at 4 Hz for CBCT imaging. Typical x-ray settings with Philips Allura were 80 kVp with 20 mA at 2 Hz for digital subtraction angiography imaging, 75 kVp with 4 mA at 15 Hz for fluoroscopy, and 120 kVp with 250 mA at 60 Hz for CBCT imaging. Nuclear acquisitions with IXSI used a 140 keV ± 15% photopeak window.

### Study End Points

The primary end points of this first-in-human study were safety and feasibility, assessed using four metrics: tracking of adverse events, a safety and feasibility questionnaire completed by personnel (radiologists and technicians), a technical performance review, and tracking the additional x-ray radiation dose. Details regarding these measures and their interpretation can be found in Appendix S1. The secondary end points related to clinical quality of the images acquired with the hybrid C-arm scanner for radioembolization guidance were not evaluated in this study.

## Results

### Participant Characteristics

**Figure 2:**
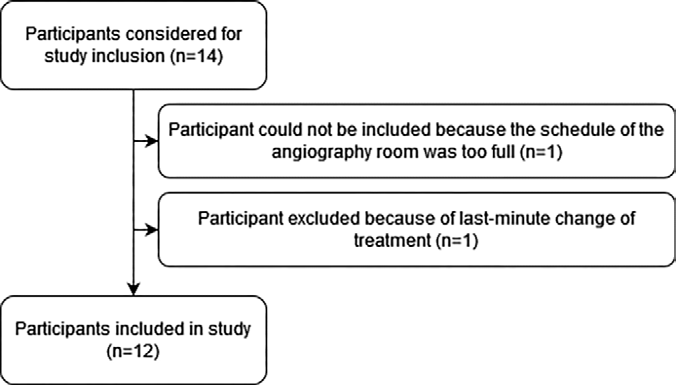
Flow diagram details the participant selection in the study.

Twelve participants (median age, 67 years [range: 37–78 years]; nine [75%] male, three [25%] female) were included in the study (Fig 2). The addition of participants was not required for any cohort. Table 1 provides baseline characteristics of the study participants. Injection positions included whole liver, lobar, segmental, and superselective. No preparatory embolization was performed during the work-up procedure. Nine planar acquisitions of the lung and liver region were planned, but only six were correctly acquired because of operator mistakes: the acquisitions were accidentally not made in one participant, only anterior (and not posterior) views were acquired in another participant, and the measurement was performed before all activity was injected in another participant. All other imaging steps were completed as planned.

**Table 1:**
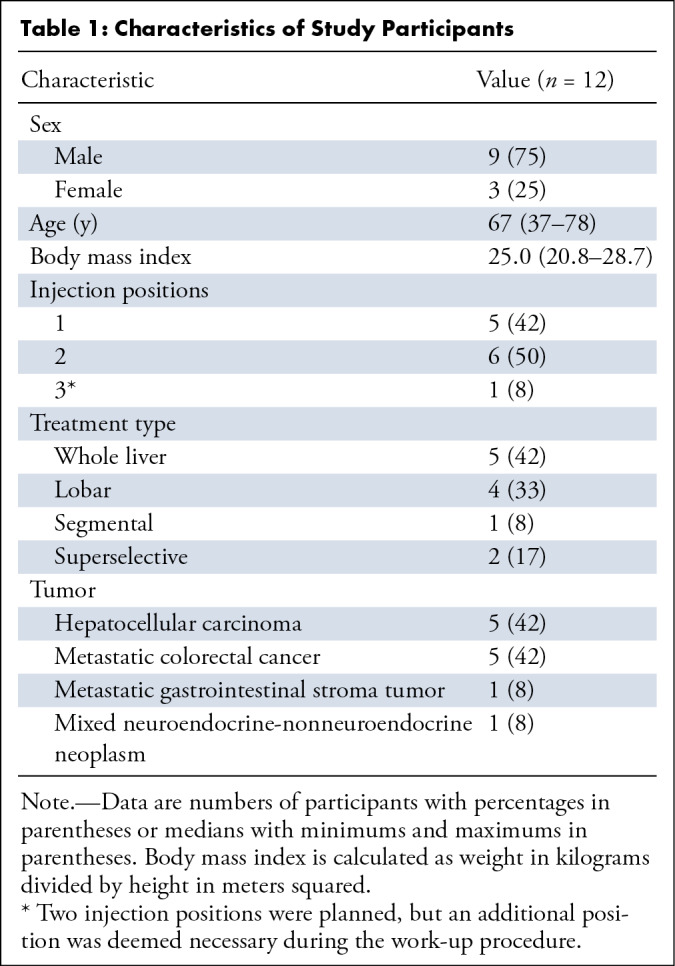
Characteristics of Study Participants

### Occurrence of Adverse Events

**Figure 3:**
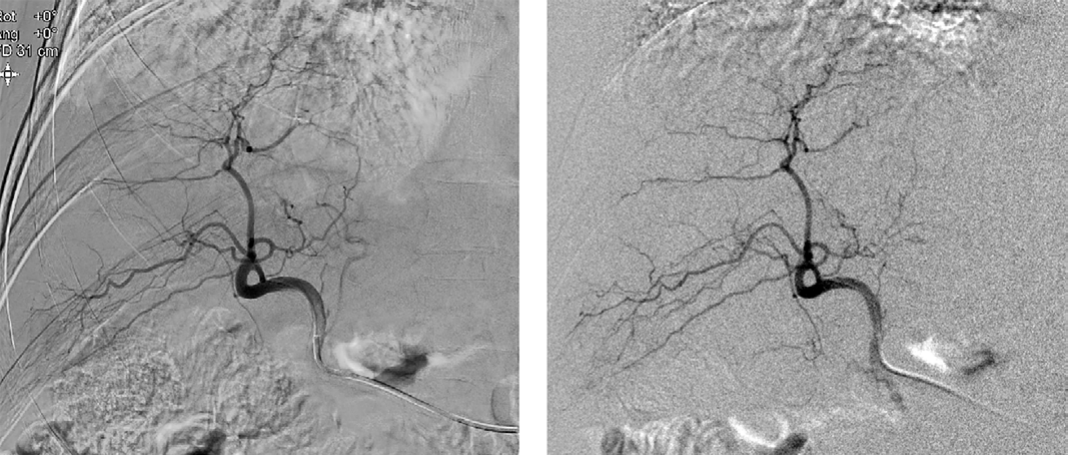
Images show comparison between the digital subtraction angiography quality made by the clinical fluoroscopy and cone-beam CT scanner (Philips Allura Xper FD20, left) and the hybrid C-arm scanner (right) in a 62-year-old male participant. The clinical system acquired better-quality images because it used a higher x-ray beam strength, had an antiscatter grid, and had a dedicated noise reduction system (ClarityIQ; Philips).

One adverse event occurred during the study (grade 1 amnesia: transient, mild, no intervention required; the hypothesis of the interventional radiologist was that a small blood clot might have entered the circulation during the procedure, but because the amnesia quickly resolved, no additional investigation was carried out), but this was not attributed to the hybrid C-arm scanner. No technical failures occurred. Specifically, repositioning of the catheter after table rotation into the hybrid C-arm scanner was not required, no collisions between the hybrid C-arm scanner and the participants occurred, all participants fit within the possible C-arm gantry orbits, no repeated rotations of the SPECT/CBCT scan were required, and it was possible to confirm the catheter position with the hybrid C-arm scanner in all procedures (see Fig 3 for a comparison of digital subtraction angiography quality).

### Radiologist Assessment of Safety and Feasibility

Interventional radiology personnel considered the use of the hybrid C-arm scanner safe and feasible in all procedures. However, the additional time spent in the intervention room for hybrid imaging using the hybrid C-arm scanner was perceived as long. Table 2 shows the duration of the imaging steps with the hybrid C-arm scanner. The median total additional time (for participants with all imaging steps performed) was 64 minutes (range: 55–77 minutes).

**Table 2:**
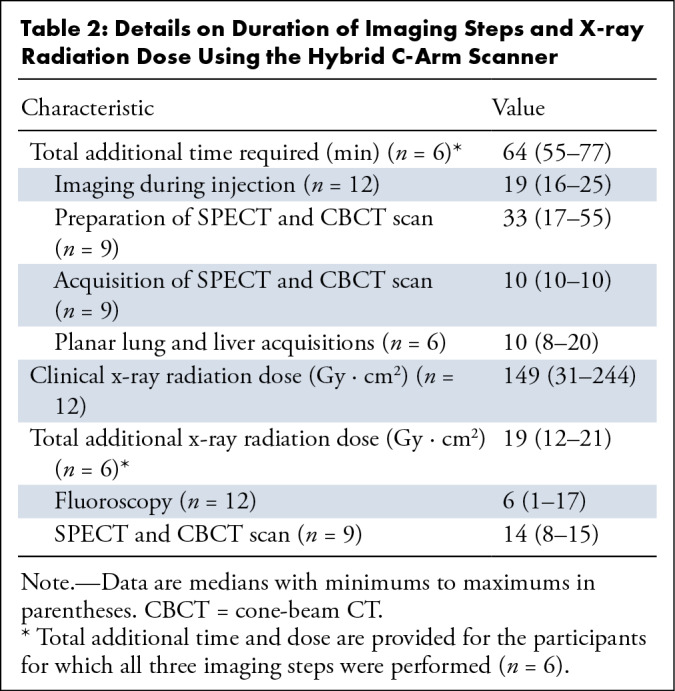
Details on Duration of Imaging Steps and X-ray Radiation Dose Using the Hybrid C-Arm Scanner

### Additional Radiation Dose

Table 2 provides the additional x-ray radiation dose administered by the hybrid C-arm scanner for the two imaging modes together with the x-ray radiation dose that was administered during the work-up procedure (both measured through dose area product meters). The median additional x-ray radiation dose (for participants with all imaging steps performed) was 19 Gy · cm^2^, which was 12.8% (149 Gy · cm^2^) of the median work-up radiation dose. The radiation dose from radioisotopes was not included in the comparison since participants would receive this dose regardless of the device used for treatment guidance.

### Representative Images

**Figure 4:**
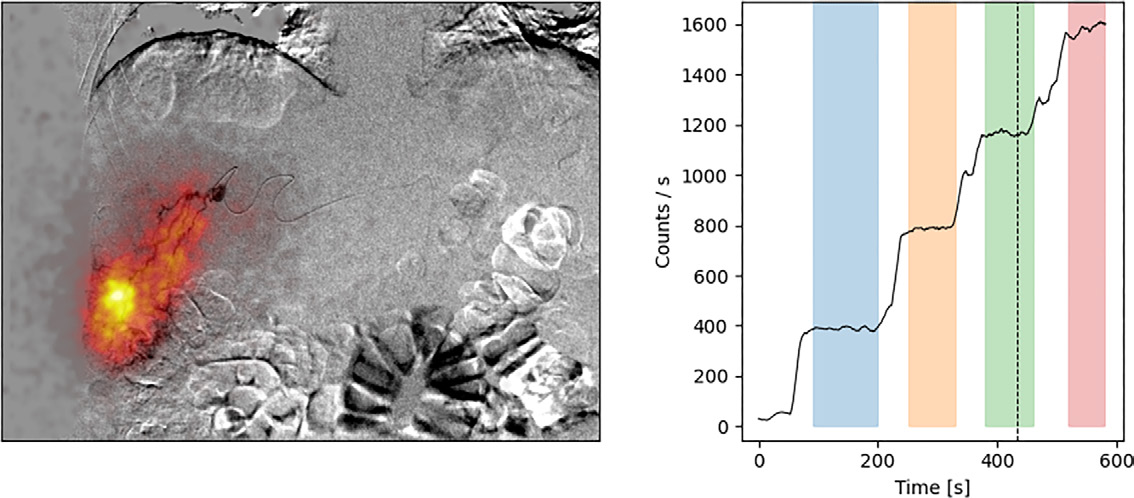
Real-time scintigraphic images acquired during the injection of technetium 99m–macroaggregated albumin in a 74-year-old male participant. Left: A still image of the scintigraphy images (color) overlaid onto the digital subtraction angiography image (gray scale). Right: Graph of the number of counts measured in the scintigraphic image, which serves as a measure of the amount of radioactivity. The four jumps in the measured counts correspond to the four syringes of approximately 25 MBq technetium 99m–macroaggregated albumin each. Dashed line on right panel indicates the frame in time that is visualized in the left panel.

Figure 4 shows a still image from a real-time hybrid imaging time series of a ^99m^Tc-MAA injection. The Movie shows time series. These visuals show the buildup of radioactivity in the tumor upon injection of four ^99m^Tc-MAA syringes together with the liver vasculature and catheter position.

**Movie:** Real-time scintigraphic images acquired during the injection of ^99m^Tc-MAA in a 74-year-old male participant. Left: the scintigraphy images (color) overlaid onto the digital subtraction angiography (DSA) image (grayscale). Right: the number of counts measured in the scintigraphic image which serves as a measure of the amount of radioactivity. The four jumps in the measured counts correspond to the four syringes of approximately 25 MBq each.

**Figure 5:**
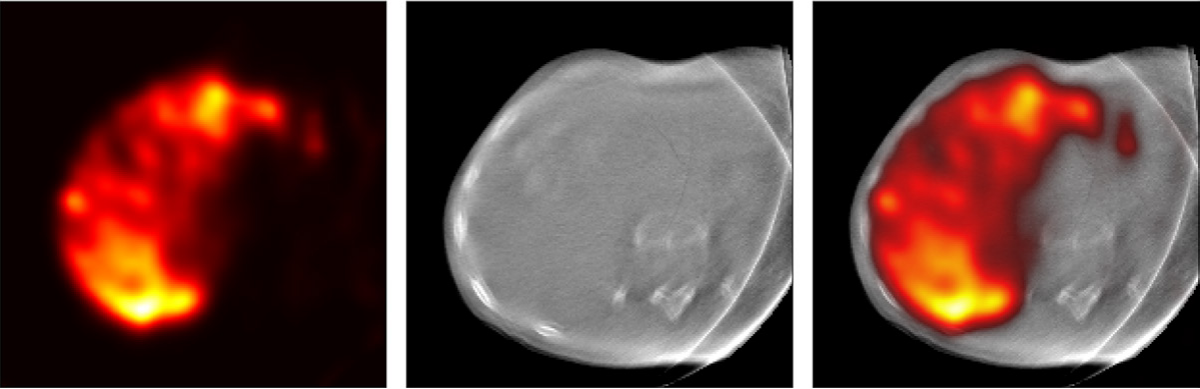
Representative images of a hybrid SPECT (left, right) and cone-beam CT (middle, right) scan obtained with the hybrid C-arm scanner in the intervention room almost immediately after injection of all technetium 99m–macroaggregated albumin in a 52-year-old female participant.

Figure 5 shows images of a SPECT/CBCT acquisition with the hybrid C-arm scanner. The images illustrate that the hybrid C-arm scanner can retrieve three-dimensional radioactivity distribution images in the demanding environment of the intervention room.

**Figure 6:**
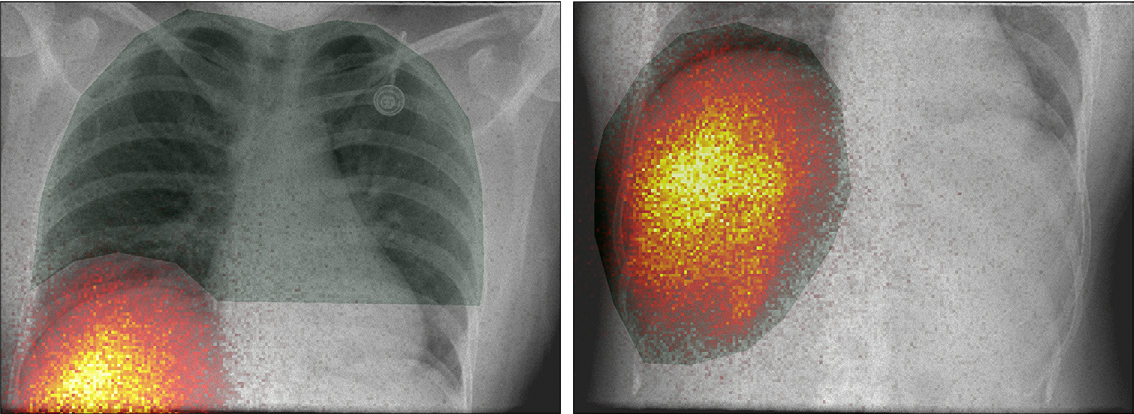
Hybrid planar images of the lung (left) and liver (right) regions acquired with the hybrid C-arm scanner in the intervention room almost immediately after injection of all technetium 99m–macroaggregated albumin in a 37-year-old female participant. The scintigraphic images are shown in color, the fluoroscopic images in gray scale, and the applied regions of interest in transparent green. The scintigraphic images may be used to measure the relative fraction of radioactivity in the lung region, which is an important measure in radioembolization for treatment planning. The fluoroscopic image may be used to make delineations of low-count regions (such as the lung in this participant).

Figure 6 shows representative images of a planar lung and liver acquisition with the hybrid C-arm scanner in the intervention room. The measurement consisted of separate acquisitions of the lung and liver regions. Masks were drawn on the lung region using the fluoroscopic image as a reference and on the liver region using the scintigraphic image as reference. These images demonstrate that the hybrid C-arm scanner may be used for measurements of the radioactivity fraction present in the lung region, which is an important criterion for safety evaluation of radioembolization treatment planning.

## Discussion

The introduction of new technology in the intervention room is not without challenges. Nevertheless, this first-in-human study of 12 participants showed that the introduction of a novel hybrid C-arm scanner into clinical practice was safe and feasible during the work-up procedure of hepatic radioembolization. There were no adverse events attributed to use of the hybrid C-arm scanner, and the additional radiation dose per participant was low (median, 19 Gy · cm^2^). The images obtained during the procedures further demonstrated the potential applications of the device for guidance of interventions involving radionuclides.

Interventional radiology personnel in our institute requested to shorten the time required for hybrid imaging to align with the busy schedule of the intervention room. In this study, much time was spent on preparing for SPECT/CBCT imaging (median, 33 minutes, minimum: 17 minutes, maximum: 55 minutes), of which most time was spent on the alignment of the patient bed with the hybrid C-arm scanner for safety purposes. Efforts are underway to shorten this preparation time.

Radioembolization was selected as the first clinical application of the hybrid C-arm scanner due to the potential benefits. For example, with in-room SPECT and lung radioactivity measurements, the work-up procedure may be merged with the subsequent treatment in a single-session procedure (9), decreasing costs, improving patient comfort, potentially improving treatment outcome (when the catheter can stay in place), and in some cases lowering radiation dose (treatment work-up is not required anymore when the catheter can stay in place). Requirements for a single-session procedure, such as the clinical evaluation of image quality, fast image reconstruction, and fast image analysis software, are subjects of current investigations.

One of the most evident advantages of using the hybrid C-arm scanner for a single-session treatment would be requiring the patient to visit only once, requiring only one catheterization, and decreasing the total procedure time. It is difficult to make a direct comparison between the conventional and proposed single-session protocol since there are some assumptions involved and the device is under active development. Nevertheless, we will provide some insights. Table S1 shows the durations of several steps of the radioembolization workflow (with median or estimated values). We found that the estimated total duration of all procedures combined in the conventional workflow was 305 minutes, while the duration of the workflow using the hybrid C-arm scanner was 169 minutes.

In the presented study, the hybrid C-arm scanner was used in conjunction with the Philips Allura system for treatment guidance. There are two limitations that hamper stand-alone use of the current version of the hybrid C-arm scanner. First, in current clinical practice, a fast breath-hold contrast-enhanced CBCT scan is frequently made. The hybrid C-arm scanner is currently unable to rotate fast enough to acquire such scans because of the weight of the gamma camera. Second, the power of the x-ray tube of the hybrid C-arm scanner is lower than that of the clinical scanner, which may be a problem in longer procedures or for larger patients. Efforts to decrease the gamma camera weight and size to allow for fast rotations are underway.

Six of nine planar acquisitions were performed correctly, while three were invalid due to operator errors (the first three acquisitions of nine). We attribute these errors to two factors. First, the acquisitions were made by medical physicists who had little experience working in angiography rooms. Second, the software that controls the hybrid C-arm scanner does not yet contain the easy-to-follow workflows that are present in commercial scanners. With more experience and implementation of workflows in the operating software, we expect that the room for error will be minimal.

One of the challenges involved in changing the radioembolization workflow is in the potential embolic effects of the particles, which can alter the flow dynamics. Although for ^99m^Tc-MAA the embolic effects have never been quantified, it has been shown for ^90^Y and holmium 166 microspheres that saturation can occur when many particles are injected (10,11). To ensure that the presence of ^99m^Tc-MAA particles has little influence on the ^90^Y microsphere distribution, we will in a planned single session be conservative in the number of ^99m^Tc-MAA particles injected.

Multiple workflows are possible for a single-session radioembolization protocol. With a single injection position, the catheter can stay in place between ^99m^Tc-MAA and ^90^Y injections. With multiple injection positions, we aim to first inject all ^99m^Tc-MAA, visualize, and afterward inject all ^90^Y. This option mimics the current two-step protocol and hence gives no complications regarding potentially altered flow dynamics. In case of two injection positions, the catheter can remain in the same location for one position. One future option for multiple injection positions (currently not planned for investigation) could be to leave the catheter in place for each injection position. This method may have the advantage of potentially being faster due to fewer catheter movements, but there may be differences in the flow dynamics between injection sites compared with the current two-step protocol.

In conclusion, this first-in-human study demonstrates the safety and feasibility of using the hybrid C-arm scanner for interventional hybrid fluoroscopy and nuclear imaging. Larger clinical trials are needed to validate these preliminary findings. Although the current study investigated its use in individuals undergoing hepatic radioembolization, the hybrid C-arm scanner may be used in various interventional procedures, such as radioguided surgeries, biopsies, and intra-arterial radionuclide therapy. This study represents the first step in clinical implementation of this hybrid C-arm scanner to guide interventional procedures, improve efficiency, and potentially improve patient care.
